# Implementation of the HACCP System for Apple Juice Concentrate Based on Patulin Prevention and Control

**DOI:** 10.3390/foods12040786

**Published:** 2023-02-13

**Authors:** Shuaishuai Duan, Fengjuan Liu, Qiaomei Qin, Qinlan Jia, Xiaoqian Cao, Zhenyu Hua, Yingying Fan, Cheng Wang

**Affiliations:** 1 College of Life Science and Technology, Xinjiang University, Urumqi 830049, China; 2 Key Laboratory of Agro-Products Quality and Safety of Xinjiang, Laboratory of Quality and Safety Risk Assessment for Agri-Products (Urumqi), Key Laboratory of Functional Nutrition and Health of Characteristic Agricultural Products in Desert Oasis Ecological Region (Co-Construction by Ministry and Province), Ministry of Agriculture and Rural Affairs, Institute of Quality Standards & Testing Technology for Agri-products, Xinjiang Academy of Agricultural Sciences, Urumqi 830091, China; 3 Department of Agriculture and Forestry Science and Technology, Weinan Vocational & Technical College, Weinan 714026, China

**Keywords:** apple juice concentrate, HACCP, patulin, critical control points

## Abstract

Patulin (PAT) is a toxic secondary metabolite produced by *Aspergillus* sp. and *Penicillium* sp., which acts as a contaminant of most apples and their products. The internationally recognized HACCP system is selected as the theoretical basis to more effectively reduce the PAT in apple juice concentrate (AJC). Through field investigation of apple juice concentrate (AJC) production enterprises, we collected 117 samples from 13 steps of AJC production, including whole apple, apple pulp, and apple juice. PAT contents were analyzed via high-performance liquid chromatography (HPLC) and compared with samples from the different production processes. The result demonstrated that the PAT content was significantly (*p* < 0.05) influenced by five processes, receipt of raw apples, sorting of raw apples, adsorption step, pasteurization, and aseptic filling. These processes were determined as the CCPs. Monitoring systems for maintaining CCPs within acceptable limits were established, and corrective actions were proposed in case a CCP was surpassed. Based on the above-identified CCPs, critical limits, and control methods (corrective actions), a HACCP plan related to the production process of AJC was established. This study provided important guidance for juice manufacturers wishing to effectively control the PAT content in their products.

## 1. Introduction

Patulin (PAT), a toxic metabolite produced by certain species of *Aspergillus* sp. and *Penicillium* sp., can be found in apples and apple products and occasionally in other foodstuffs, such as pears, apricots, peaches, kiwi, wheat, and corn [1,2]. Apple juice is a major source of PAT intake in the human diet [3]. From 2008 to 2017, the global export volume of apple juice exceeded 2.0 million tons, and the import volume exceeded 1.8 million tons [4]. When apple juice is contaminated by PAT, the economic impact is severe. As PAT is stable in acidic conditions and is resistant to heat, it remains present even after food processing. Assessment of the health risks posed by PAT to humans suggests that PAT consumption can cause acute symptoms such as pulmonary congestion, edema, ulceration, intestinal hemorrhage and inflammation, epithelial cell degeneration, and gastrointestinal and kidney damage [5]. It may also cause chronic neurotoxic, immunotoxic, genotoxic, and teratogenic effects in rodents [6]. Therefore, several countries and organizations have set maximum limits for PAT in food to control the health risk. The US Food and Drug Administration [7] limit PAT in pure and blended apple juice to 50 μg/L. The EU [8] set the maximum allowable level at 50 μg/kg in apple juice and its derivative products, 25 μg/kg in solid apple products, and 10 μg/kg in apple products intended for infants and young children. The Joint FAO/WHO Expert Committee on Food Additives [9] set a maximum daily intake of 0.4 mg/kg body weight/day for PAT. In China, standards for mycotoxins in food established by the Ministry of Health of the People’s Republic of China [10] set the maximum level for PAT in apple products and hawthorn products at 50 μg/ kg.

Surveys investigating the level of PAT contamination have been conducted in many countries. In Turkey, İçli [11] evaluated PAT in apple sour and found that the average level of PAT was 100–200 μg /kg in 13 out of 39 samples, while two samples ranged from 1000 to 1500 μg/ kg. In Spain, PAT concentrations ranged from 0.7 to 118.7 μg/L [12]. In China, Yuan et al. [13] detected apple products purchased from supermarkets and stores in northeastern China and found that more than 16% of the 95 samples (including apple juice, baby food, fruit juice concentrate, and conforming juice) contained PAT above 50 μg/kg. In Taiwan, China, Lien et al. [14] analyzed the content of PAT in imported food products and found that PAT was still detected in apple juice and apple-flavored beverages at levels higher than the permitted standard of 50 μg/kg.

Hazard analysis and critical control points (HACCP) is a preventive approach used to control food processing by identifying hazards in the food production process, controlling hazards, and reducing risks [15]. As food safety issues continue to emerge, the worldwide use of HACCP systems is becoming increasingly widespread, and the prevalence of foodborne illnesses is decreasing. Tomasevic Igor et al. [16] found that since the implementation of the HACCP system, the percentage of fresh meat and meat product enterprises in Serbia that did not meet the necessary standards dropped from 18.6% to 8.3%, and the concentration of nitrite content in meat product decreased by 43%. The FDA [17] has published an industry guide for juice production which aims to help juice companies identify possible hazards in their products and control the occurrence of hazards in a timely manner. Minor and Parrett [18] found that the implementation of the "Hazard Analysis and Critical Control Point (HACCP): Safe and Sanitary Processing and Importation Procedures for Fruit Juices" reduced the prevalence of annual foodborne illnesses, with 462–508 fewer cases reported. In China, both Zhu [19] and Hua [20] established a HACCP system for apple juice based on traditional hazards. However, in rare cases, the HACCP system is used to control mycotoxins.

China is the largest apple juice concentrates producer and supplier in the world, accounting for 60% of the global trade volume [21]. When relevant products are contaminated by PAT, there are many economic impacts and food safety issues. HACCP is a well-established methodology for minimizing food risks associated mainly with microbial hazards. It is not so widely applied to the management of toxins and other chemical risks. In this study, we surveyed the AJC processing enterprise, took samples from possible risk points, and measured the content of PAT, aiming to provide data support for the identification of critical control points (CCPs) and establish a HACCP system on PAT prevention and control. The implementation of the HACCP system is of great significance to reduce the safety risk of PAT in AJC, and we wish to promote a broader application of the HACCP philosophy to the control of toxin-related hazards in the food industry.

## 2. Materials and Methods

### 2.1. Reagents

Citric acid and pectinase were supplied by Beijing Solarbio Technology Co., Ltd. (Beijing, China). LC-MS grade acetonitrile was purchased from Thermo Fisher Scientific (Waltham, MA, USA). HPLC-grade water was generated by Watson Group Ltd. (Hong Kong, China). Molecular sieve 4 Å was bought from Aladdin Reagent Co. Ltd. (Shanghai, China), and C18 was purchased from Varian China Ltd. (Beijing, China). 

### 2.2. Sampling

All samples were collected from Aksu Tianshan Shenmu fruit industry development Co., Ltd. A total of 117 samples were obtained, including fruits, pulp, and juice from 13 steps of AJC production. The samples of apple fruit after cutting and pulping were put in plastic boxes, pulp samples collected from production processing were put in plastic boxes, and liquid samples were stored in plastic bottles. All samples were stored at −20 °C for subsequent experiments.

### 2.3. PAT Analysis

The sample preparation was performed as outlined by Zhang [22], in which the LOD and LOQ in the apple juice matrix were 0.62 μg/L and 2.09 μg/L, PAT standard solutions at concentrations of 10 μg/L, 50 μg/L, and 100 μg/L, respectively, were added to the apple samples, the recovery rate in apple juice samples was approximately 98.70% to 108.00%. The linearity obtained at R^2^ > 0.9995 for PAT ranged from 0 to 1000 μg/L as revealed by HPLC analysis, demonstrating the linearity of the method over the entire calibration range.

For liquid samples, 10 mL of the liquid sample and 10 mL of extraction solution (10 mmol/L citric acid acetonitrile solution) were absorbed successively into a 50 mL centrifuge tube and shaken vigorously for 3 min (Note: samples collected before the enzyme digestion process should be treated with pectinase in advance and overnight). Then, 4 g of 4 A molecular sieve (dried at 400 °C for 3 h, immediately transferred to a desiccator and cooled to room temperature before use) and 1 g NaCl were added, shaken vigorously for 1 min, and centrifuged at 9000 rpm/min for 5 min. Next, 2 mL of supernatant was transferred to a 15 mL centrifuge tube. Next, 60 mg of purifying agent C18 was added, the mixture was vortexed for 1 min, and centrifuged at 10,000 rpm for 3 min. The supernatant was passed through a 0.22 μm PTTE filter membrane.

For solid samples, 2.5 g of homogenized apple samples and 2.5 mL of extraction solution were added to a 50 mL centrifuge tube and treated with pectinase overnight. Then, 1 g of molecular sieve and 45 mg of C18, and 0.25 g of NaCl were added; the other extraction procedures were constant with the liquid samples above. 

The final extraction samples were determined by high-performance liquid chromatography (HPLC, Waters, Milford, MA, USA). An Xselect HSS T3 column (Waters, 5 μm, 4.6 × 250 mm) was used; the column temperature was 40 °C, and the samples were injected at a flow rate of 1 mL/min under the condition of mobile phase: acetonitrile: water = 77:23 (*v*/*v*), with a sample volume of 10 μL each time.

### 2.4. Statistical Analysis

The Excel 2016 software was used to calculate descriptive statistics of PAT content obtained from HPLC. Dates were presented as mean ± standard deviation (SD). Statistical analysis was performed using the Analysis of Variance (ANOVA) with SPSS 27.0 software. Differences at *p* < 0.05 were considered significant. Data from different processes were plotted as line graphs using Origin 2019 software to reflect the changes in PAT content in the samples at different processing stages.

## 3. Results and Discussion

### 3.1. The Production Process of AJC and Samples Source

**Figure 1:**
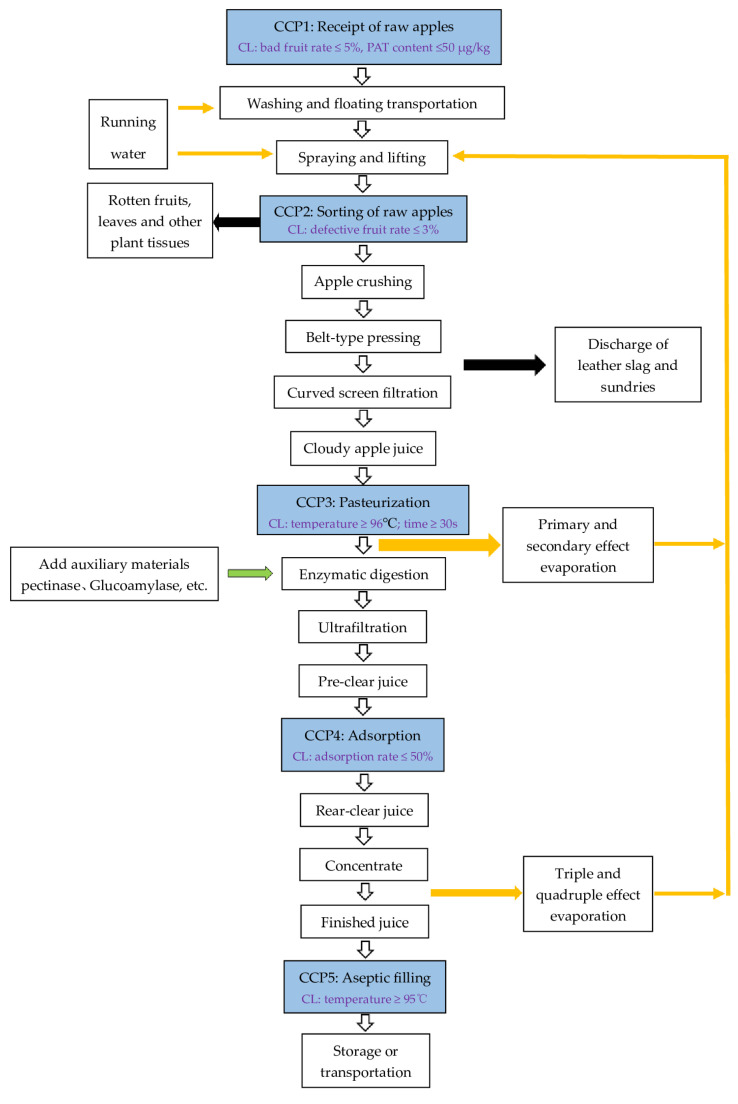
Process flow chart for apple juice concentrate. Abbreviations: CL—critical limits. (Processes in blue box represent critical control points, processes indicated by black arrow represent waste, processes pointed by yellow arrow represent water cycle, and processes indicated by green arrow represent auxiliary material addition).

The main production process of AJC is shown in Figure 1. The samples were collected from the following points: receipt of raw apples (RRA), sorting of raw apples (SRA), belt-type pressing (BTP), cloudy apple juice (CAJ), pasteurization (PAS), enzymatic digestion (ED), ultrafiltration (UF), pre-clear juice (PCJ), adsorption (AD), rear-clear juice (RCJ), concentration (CONC), finished juice (FJ), and aseptic filling (AF).

### 3.2. Analysis of PAT Content Changes

**Figure 2:**
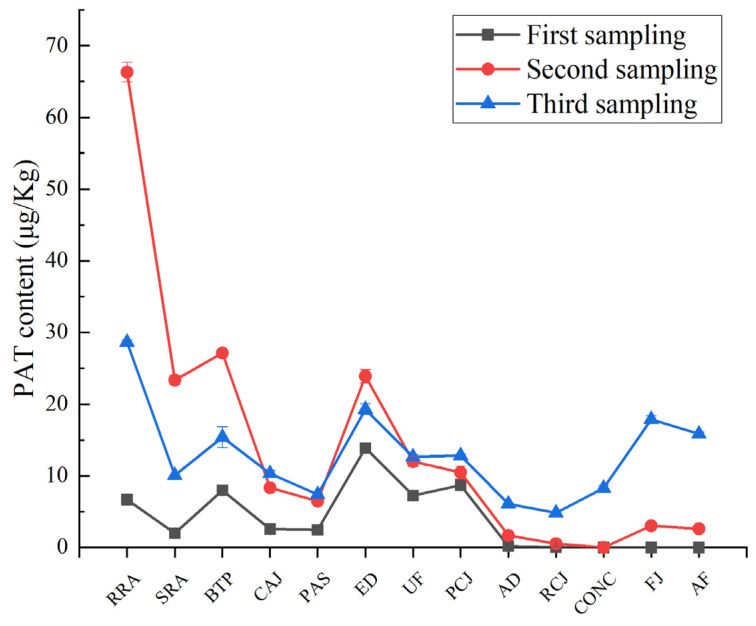
PAT content changes in three samplings. Abbreviations: RRA—receipt of raw apples. SRA—sorting of raw apples. BTP—belt-type pressing. CAJ—cloudy apple juice. PAS—pasteurization. ED—enzymatic digestion. UF—ultrafiltration. PCJ—pre-clear juice. AD—adsorption. RCJ—rear-clear juice. CONC—concentration. FJ—finished juice. AF—aseptic filling.

Results from HPLC analysis (Table S1 and Figure 2) showed that the PAT content of samples is different from the AJC processing steps. The overall trend of PAT content in three groups of samples collected at different times was consistent. Samples collected for the second time were analyzed as an example. 

The highest amount of PAT in RRA was 66.33 μg/kg. After washing and sorting, the PAT content of the SRA was significantly reduced, and the PAT content was reduced to 23.37 μg/kg. Belt pressing and curved screen filtration could decrease the PAT content before the CAJ steps (8.32 μg/kg). Although pasteurization treatment showed a weak decreasing trend with regard to PAT content, enzymatic digestion caused an increase in PAT, from 6.49 μg/kg to 23.91 μg/kg. Both the ultrafiltration and adsorption reduced the PAT content, and the PAT content in AD was only 1.67 μg/kg. The PAT content in the FJ was still controlled within an acceptable range, even though concentration increased the PAT content, and the PAT content in aseptic filling finally only reached 3.03 μg/kg.

### 3.3. Hazard Analysis

From the receipt of raw apples to the storage and transportation of the final products, hazard analysis was required as part of every technical procedure to define factors that may affect the PAT content [23] (Table S2). The severity and risk will determine the significance of each hazard. In Figure 2, the changes in the PAT content in every production process were different. Table 1 shows the effect of some processing stages on PAT content in apple juice concentrate.

#### 3.3.1. Receipt of Raw Apples

When considering apples as a raw material for juice production, fruit ripeness, bruising, and decay rate seriously affects the number of toxins in the product. Toxin-producing fungi present on the fruit surface and in decayed fruit are the fundamental source of PAT in apple juice, and failure to treat these issues during juice production could increase the amount of PAT in the final product [24]. Before harvesting the raw materials, detection of PAT and fungi testing are performed for each origin at the same time; other hazard factors such as pesticide and heavy metal (lead and total arsenic) residues also require testing.

#### 3.3.2. Washing and Sorting of Raw Fruit

The content of PAT in the samples of SRA was considerably lower compared to the toxin content in the RRA combined with Table 1 and Table S1, indicating that the sorting of raw apples had a significant effect on the removal of PAT, with a reduction of 71.59%, 64.76% and 64.80% (*p* < 0.05) in the corresponding stages of the three samplings. Meanwhile, the moisture, sweetness, and acidity of apples are highly suitable for the survival of toxin-producing fungi. Therefore, strict screening and removal of rotten apples contribute significantly to controlling the content of PAT in the final product, reducing the bad fruit rate to under 5%. 

#### 3.3.3. Belt-Type Pressing

Belt-type pressing treatment had some effect on the removal of PAT from the juice and could release the PAT from apples, as demonstrated by a study by Bissessur et al. [25], in which the content of PAT in BTP increased compared to the SRA after the belt-pressing step. The pressing process must be conducted in a sanitary environment; the machine should be fully cleaned and sterilized in advance to ensure that no metal fragments or parts remain. 

#### 3.3.4. Curved Screen Filtration and Waste Emission

The curved sieve separated apple juice, apple pulp, and other waste, and the content of PAT in CAJ was reduced (*p* < 0.05), possibly due to the interception of large particulate by a curved sieve and the removal of additional apple tissue [26]. The curved screen requires timely clean-up to prevent the accumulation of waste, and a strict maintenance plan is implemented. The inspection was carried out at every fixed time interval during the production process.

#### 3.3.5. Pasteurization

Although pasteurization did not have a significant effect on the removal of PAT in first-group samples, both second and third group samples demonstrated a significant effect (*p* < 0.05). The average removal of PAT in the second and third-samples was about 25.17%.

The pasteurization process has provided some assistance in reducing PAT content [27]. Extra-high temperatures can damage the materials’ nutrients and bioactivity and affect the flavor of the final product. Appropriate pasteurization is effective for the removal of pathogens and toxins that may be present in the product and the juice production, and it facilitates them in the final product below acceptable limits [28]. It must be ensured that the sterilization temperature and time are within the appropriate range.

#### 3.3.6. Enzymatic Digestion

There was a significant increase in the amount of PAT in the ED compared to that in the CAJ, which slightly deviated from the conclusions obtained by others. It was speculated that the enzymatic reaction released PAT from the fine particulate in the cloudy juice tank, resulting in an increase in the PAT content in the ED. 

Pectinase, glucoamylase, and water and food additives were fully dissolved in water in another vat, then added to the mixing vat and fully mixed with apple juice. The feeding process was conducted in a sanitary environment, and the mixing vat was fully cleaned and sterilized in advance to ensure no pathogens remained. Additives needed to fulfill the requirements of the National food safety standard: Standards for limitation of food additives [29].

#### 3.3.7. Ultrafiltration

During the AJC production process, UF was adopted to remove insoluble solids, colloidal particles, and a few microorganisms prior to evaporation and concentration, while PAT, as a small molecule, could be filtered out along with other macromolecules [30]. This resulted in a certain decrease in PAT content in the product after ultrafiltration. Regular cleaning was carried out during the production process, ensuring no pathogens and toxins remained.

#### 3.3.8. Adsorption Step

The study showed that the adsorption step had a significant effect on the reduction of PAT content, and the changes in PAT during the adsorption step in the three samplings were 6.08 μg/kg, 8.82 μg/kg, 5.59 μg/kg, and the toxin adsorption rates were 90.88%, 84.08%, and 47.86%. However, there was no replacement or cleaning of the relevant materials in this step by the production enterprise during our three samplings. Additionally, the adsorption capacity of the adsorption column approached saturation over time, causing the adsorption rates to decrease [31] gradually. Therefore, regular cleaning or replacement of the adsorption material plays a significant role in reducing PAT content in the final product, and no pathogenic toxins should remain on the core material.

#### 3.3.9. Concentration

The purpose of concentration is to remove excess water and increase the concentration of apple juice; this is why the PAT content in the FJ increases.

#### 3.3.10. Aseptic Filling

In this study, the content of PAT was decreased by 1.81 μg/kg and 3.02 μg/kg separately in the second and third samples. Additionally, the aseptic filling can significantly decrease the concentration of PAT (*p* < 0.05, between CONC and AF). This could be a critical control point. Additionally, the packaging materials should meet the appropriate standards, and production conditions should be in line with the production permit requirements to prevent secondary contamination of the product by fungal microorganisms. They should also be able to withstand changes in temperature and humidity to protect the product from external environmental factors. It is also vital that the product containers should not come into contact with any insects, rodents, or other contaminants that may affect product quality.

### 3.4. Identification of CCPs and Critical Limit

Based on the result of hazard analysis and data analysis, the AJC production process contains five CCPs. The critical limit is the processing requirement that needs to be fulfilled at every corresponding control point. The HACCP system is applied in the present study as a preventive food safety approach to control the potential hazards of PAT appearing in the AJC.

#### 3.4.1. CCP1 Receipt of Raw Apple

While receiving raw apples, the bad fruit rate should be kept under ≤5% (for each batch of raw apple samples, 20 kg of apples are randomly taken out, in which the total weight of broken or rotten apples should be less than 1kg, or else, the corresponding batch is not up to standard), and the PAT content should be lower than 50 μg/kg. Toxin-producing fungi present on the fruit surface and in decayed fruit are the fundamental source of PAT in apple juice [24], so the most fundamental measure is to reduce the content of PAT in raw apples through the strict receiving step. Failure to treat these bad fruits could increase the accumulation of PAT in juice.

#### 3.4.2. CCP2 Sorting of Apple

After the sorting step, the apple will directly enter the crushing step. If many rotten apples are identified prior to the crushing step, the content of PAT in the downstream products may remain above the set limits. Therefore, combining manual with machine sorting of apples ensures the rate of defective fruit remains under 3% (a total of 5 kg of apples on the sorting table are randomly removed every hour; thus, the total weight of rotten apples should be less than 0.15 kg). This will effectively guarantee the safety of downstream products. The risk of fungal and bacterial contamination could be effectively reduced by cleaning the fruit surface, as well as removing moldy fruits and decaying leaves, and other plant tissues with fungal and bacterial contamination [25,32].

#### 3.4.3. CCP3 Pasteurization

In order to reduce PAT and kill related fungi in pasteurization steps, sterilization temperature ≥96 °C and sterilization time ≥30 s should be ensured, but extra-high temperature or extra-long pasteurization may negatively affect product quality. Raiola et al. [33] found that the removal of PAT in artificially contaminated apple juice via secondary sterilization was about 62.62 ± 2.53% (from 50 to 19.10 μg/L) via simulated experiments.

#### 3.4.4. CCP4 Adsorption Step

The adsorption step plays a major role in reducing the PAT content [34]. When the adsorption efficiency is less than 50%, the core material should be cleaned or replaced in time. The decrease in the interception capacity of the adsorption column approaches saturation over time. Additionally, the accumulation of PAT increases with the increase in AJC production over time, resulting in an increase in PAT content in the final product at the third sampling.

#### 3.4.5. CCP5 Aseptic Filling

During aseptic filling, the filling head temperature should be ≥ 95 °C to prevent secondary contamination of the product by fungal microorganisms. Additionally, the packaging materials should meet the appropriate standards, and production conditions should be in line with the production permit requirements.

### 3.5. Related Control Methods Based on CCPs

In summation, the processes, including receipt of raw apples, sorting of raw apples, pasteurization, adsorption step, and aseptic filling, are the critical control points. If the PAT value exceeds the standard limit value of 50 μg/kg, the filling of the product into the tank and the subsequent finished product should be stopped, and prompt action should be taken to determine the cause of the excess PAT and treat the product to reduce the PAT content to an acceptable level.

Based on the identified CCPs, the risk of fungal and bacterial contamination in raw apples could be effectively reduced through stricter receiving processes, cleaning the fruit surface, and removing moldy fruits and decaying leaves, and other plant tissues with fungal and bacterial contamination [25]. It is worth noting that although low temperatures inhibit the ability of toxigenic fungi to produce toxins, apples that have been stored for too long are more susceptible to fungal contamination, leading to a higher risk of increased PAT content during production [35]. In a study by Ma et al. [36], methyl thujate inhibited the growth of *P. expansum* mycelium and reduced PAT production in postharvest fruits. Zhang et al. [37] found that ClO2 fumigation of apples infected with *P. expansum* significantly suppressed the diameter of apple spots, inhibiting the growth of mycelium and spore germination. Methyl thujate treatment and ClO_2_ fumigation may be helpful in reducing PAT after apple sorting. The selection of adsorption core materials with higher efficiency and stronger capacity can reduce the frequency of cleaning core materials and increase the efficiency of production enterprises. Assaf et al. [38] showed that the PAT degradation rate reached 50% after sterilization of AJC semifinished products with ascorbic acid addition, which was protected from light under low-oxygen conditions. In addition to the most commonly used methods of adsorption and sterilization for apple juice, ozonation [39] and UV treatment [40] for apple juice samples showed a significant reduction in PAT content in the final product; meanwhile, the nutritional value and sensory product were within acceptable limits. 

PAT is a secondary metabolite of fungi. Thus, inhibiting the growth activity of fungi and reducing its toxin-producing capacity or degrading it via physical, chemical, and biological methods could reduce the PAT content introduced to AJC through different steps, successfully protecting consumer safety [41].

### 3.6. Establishment of HACCP Plan

A reasonable and applicable HACCP plan could improve the management level of the food plant, enhance the safety awareness of employees, and reduce the incidence of food safety accidents. Thus, based on the above-identified CCPs, critical limits, and control methods, a HACCP plan related to the production process of AJC was established. As shown in Table 2, the supervision, record, and verification measures for each CCP were also listed. In order to effectively implement the HACCP plan for the quality of AJC, it is often necessary to set up a team including the person in charge of the enterprise, professional personnel, and relevant operators in charge of product quality control, production management, health management, product development, procurement, storage, and equipment maintenance, etc.

## 4. Conclusions

Through a survey and subsequent data analysis, this study applied HACCP principles to analyze the hazards of each process during AJC production and found that receipt of raw apples, sorting of raw apples, adsorption, pasteurization, and aseptic filling, are the five CCPs in the AJC production process. Based on the identified CCPs and the corresponding appropriate prevention and control methods, a HACCP plan was developed, which has a strong application value for the quality of AJC, especially regarding the effective control of PAT contamination.

## Figures and Tables

**Table 1:** Effect of processing stages on PAT content in apple juice.

Sampling Points	First Sampling		Second Sampling		Third Sampling	
	PAT Content (Mean ± SD, μg/kg)	Reduction Rate (%)	PAT Content (Mean ± SD, μg/kg)	Reduction Rate (%)	PAT Content (Mean ± SD, μg/kg)	Reduction Rate (%)
Receipt of raw apples (RRA)	13.89 ± 0.74	0	66.34 ± 2.95	0	28.64 ± 0.33	0
Sorting of raw apples (SRA)	3.97 ± 0.28	71.42	23.37 ± 1.45	64.77	10.30 ± 0.56	64.04
Pasteurization (PAS)	2.46 ± 0.19	7.87	6.49 ± 0.40	22.00	7.41 ± 0.16	28.34
Adsorption (AD)	0.61 ± 0.02	90.88	1.67 ± 0.21	84.08	6.09 ± 0.21	47.86
Aseptic filling (AF)	/	/	1.62 ± 0.12	52.92	7.54 ± 0.79	28.60

**Table 2:** HACCP plan for apple juice concentrate.

CCPs	Critical Limit Values for Preventive Measure	Supervision				HACCP Records	Verification
		What to Monitor	How to Monitor	Monitoring Frequency	Who Will Monitor		
CCP 1: Receipt of raw apples	Bad fruit rate is controlled under 5%	Bad fruit rate	Visual inspection of bad fruit	Each batch of raw fruit	Quality control people	1. Official test report 2. Fruit raw material inspection records 3. Correction records	Review the test report or acceptance records
CCP 2: Sorting of raw apples	The rate of defective fruit is controlled under 3%	Defective fruits	Visual defective fruits	Hourly	Process quality control people	1. Process quality control records 2. Correction records	The workshop supervisor and the process quality controller review the records daily to verify the corrective measures.
CCP 3: pasteurization	Sterilization temperature is ≥ 96 °C.Sterilization time is ≥30 s.	1. Sterilization temperature 2. Sterilization time (product flow)	1. Measuring temperature 2. Observe the flow of products	Hourly view of sterilization temperature and product flow	Sterilization process operators	1. Filling process record sheet 2. Correction records	1. Take finished product samples every hour for microbiological testing 2. regular maintenance and check and corresponding monitoring instruments 3. Review records
CCP 4: Adsorption step	PAT adsorption efficiency is more than 50%	PAT removal efficiency between pre-cleaning and post-cleaning tanks	Determination of PAT content in pre-cleaning and post-cleaning tanks	Weekly	Quality inspector	1. Quality inspection department test report 2. Correction records	1. The director of the quality inspection department 2. Relevant testing personnel review the records weekly to verify the corrective measures 3. Reviewing the test reports.
CCP 5: Aseptic filling	Filling head Temperature is ≥95 °C	Filling head Temperature	Measuring temperature	Check filling temperature every hour	Filling process operators	1. Filling process record sheet 2. Correction records	1.Take finished product samples every hour for microbiological testing 2. Regularly overhaul and calibrate filling machines and corresponding monitoring instruments 3. Review records

## Data Availability

The data are available from the corresponding author.

## Supplementary Materials

The following supporting information can be downloaded at: https://www.mdpi.com/article/10.3390/foods12040786/s1**,** Table S1: PAT content of AJC samples from different processing points; Table S2: Hazard analysis for apple juice concentrate
