# Supplementary material for: Implementation of the HACCP System for Apple Juice Concentrate Based on Patulin Prevention and Control
Source: Foods. 2023 Feb 13;12(4):786. doi: 10.3390/foods12040786 (PMC9956176; doi:10.3390/foods12040786)
Supplement: Supplementary file 1 [file foods-12-00786-s001.zip › foods-2180115-supplementary.pdf]

Supplementary Materials

Table S1: PAT content of AJC samples from different processing points.

Sampling points	PAT content (Mean ± SD, μg/kg)		
	First sampling	Second sampling	Third sampling
Receiving of raw apples (RRA)	13.89±0.74 a	66.34±2.95 a	28.64±0.33 a
Sorting of raw apples (SRA)	3.97±0.28 e	23.37±1.45 c	10.30±0.56 e
Belt-type pressing (BTP)	7.99±0.37 c	27.14±1.33 b	15.45±0.80 c
Cloudy apple juice (CAJ)	2.67±0.38 f	8.32±0.21 e	10.34±0.40 e
Pasteurization (PAS)	2.46±0.19 f	6.49±0.40 f	7.41±0.16 f
Enzymatic digestion (ED)	8.69±0.25 b	23.58±0.97 c	19.29±0.92 b
Ultrafiltration (UF)	7.25±0.31 d	12.01±0.74 d	12.84±0.92 d
Pre-clear juice (PCJ)	6.69 ±0.37 d	10.49±0.92 d	11.68±0.93 d
Adsorption (AD)	0.61±0.02 g	1.67±0.21 h	6.09±0.21 g
Rear-clear juice (RCJ)	/	0.53±0.04 h	4.85±0.57 h
Concentrate (CONC)	/	1.27±0.04 h	8.35±0.39 f
Finished juice (FJ)	/	3.43±0.29 g	10.56±0.52 be
Aseptic filling (AF)	/	1.62±0.12 h	7.54±0.79 cf

Note: Values with different superscript letters indicate significant difference at $P<0.05$ in one column.

Table S2: Hazard analysis for apple juice concentrate.

Samples points	Hazard factors	Prevention and control measures
Receiving of raw apples	Biological: Pathogenic bacteria contamination	Sterilization control through the back
	Chemistry: 1. Pesticide residues;<br>2. Heavy metal (lead and total arsenic);<br>3. Patulin	1. Before harvesting the raw materials, the pesticide residues and heavy metal (lead and total arsenic) residues are tested for each batch.<br>2. Microbiological testing<br>3. Control the bad fruit rate within 5%.
	Physical: fragments, dirt, gravel and other impurities	Controlled by floating water conveying process.
Receiving of auxiliary materials	Biological: None	
	Chemical: Heavy metal (lead and total arsenic) residues exceed the standard	Evaluation of supplier qualifications, every six months by the supplier to provide product compliance report, do not meet the rejection.
	Physical: None	
Receiving of package materials	Biological: None	
	Chemistry: None	
	Physical: None	

Washing and floating transportation	Biological: Pathogenic bacteria contamination	1. Regular testing of water quality to ensure compliance with *Standards for Drinking Water Qual* (2006) requirements.<br>2. Control equipment hygiene through operational pre-requisite program (OPRP).
	Chemistry: None	
	Physical: metal fragments, dirt, gravel and other impurities	Strictly implement the operation procedures of fruit washing process
Spraying and lifting	Biological: Pathogenic bacteria contamination	1. Regular testing of water quality to ensure compliance with *Standards for drinking water quality* (2006) requirements.<br>2. Control equipment hygiene through OPRP.
	Chemistry: None	
	Physical: Metal fragments or parts	Strict maintenance plan is implemented, inspection is carried out every 4 hours during the production process, abnormalities are immediately stopped
Sorting of raw apples	Biological: Pathogenic bacteria contamination	1. Regular testing of water quality to ensure compliance with *Standards for Drinking Water Qual* requirements.<br>2. Control equipment hygiene through OPRP.
	Chemistry: Patulin	The decay rate is within 3%.
	Physical: None	
Apple crushing	Biological: Pathogenic bacteria contamination	Control of equipment hygiene through OPRP.

	Chemistry: None	
	Physical: Metal fragments or parts	Strict maintenance plan is implemented, inspection is carried out every 4 hours during the production process, abnormalities are immediately stopped
Collecting of row juice	Biological: Pathogenic bacteria contamination	1. Regular testing of water quality to ensure compliance with *Standards for drinking water quality* (2006) requirements.<br>2. Control equipment hygiene through OPRP.
	Chemistry: None	
	Physical: Metal fragments or parts	Strict maintenance plan is implemented, inspection is carried out every 4 hours during the production process, abnormalities are immediately stopped
Pasteurization	Biological: Pathogenic bacteria contamination	Strict control of sterilization temperature and time
Enzymatic digestion	Biological: Pathogenic bacteria contamination	Control of equipment hygiene through OPRP.
Concentration	Chemical: Heavy metal (lead and total arsenic)	Evaluation of supplier qualifications, every six months by the supplier to provide product compliance report, do not meet the rejection.
	Physical: None	
	Physical: None	
Ultrafiltration	Biological: Pathogenic bacteria contamination	Control of equipment hygiene through OPRP.
	Chemistry: None	

	Physical: None	
Adsorption	Biological: Pathogenic bacteria contamination	Control of equipment hygiene through OPRP.
	Chemistry: Patulin	Control by replacing or cleaning the core material
	Physical: None	
Concentration	Biological: None	Control of equipment hygiene through OPRP.
	Chemistry: None	
	Physical: None	
Aseptic filling	Biological: Pathogenic bacteria contamination	Strict control of filling room temperature
	Chemistry: None	
	Physical: None	
Labeling and	Biological: Organisms: pests, rodents	Control of equipment hygiene through OPRP.
Barrels sealing	Chemistry: None	
	Physical: None	
Storage or	Biological: None	
Transportation	Chemistry: None	
	Physical: None	

Abbreviation: OPRP, operational pre-requisite program.
